# Hypertension is associated with reduced resting‐state medial temporal lobe dynamic network flexibility in older African Americans

**DOI:** 10.14814/phy2.16084

**Published:** 2024-06-08

**Authors:** Joshua L. Gills, Darian A. Napoleon, Miray Budak, Bernadette A. Fausto, Mark A. Gluck, Steven K. Malin

**Affiliations:** ^1^ Department of Psychiatry New York University Grossman School of Medicine New York New York USA; ^2^ Department of Population Health New York University Grossman School of Medicine New York New York USA; ^3^ Center for Molecular and Behavioral Neuroscience Rutgers University‐Newark Newark New Jersey USA; ^4^ Department of Kinesiology and Health Rutgers University New Brunswick New Jersey USA; ^5^ Division of Endocrinology, Metabolism and Nutrition Rutgers University New Brunswick New Jersey USA; ^6^ New Jersey Institute for Food, Nutrition and Health Rutgers University New Brunswick New Jersey USA; ^7^ Institute of Translational Medicine and Science Rutgers University New Brunswick New Jersey USA

**Keywords:** Alzheimer's disease, cardiovascular disease, cognition, ethnicity, obesity

## Abstract

Hypertension disproportionately affects African Americans and is a risk factor for Alzheimer's disease (AD). We investigated the relationship of blood pressure (BP) with medial temporal lobe (MTL) dynamic network flexibility (a novel AD biomarker) and cognitive generalization in older African Americans. In a cross‐sectional study, 37 normotensive (systolic BP <130 mmHg, 82.5% F, 64.4 ± 4.9 years; 14.3 ± 2.1 years of education) versus 79 hypertensive (systolic BP ≥130 mmHg, 79.5% F, 66.8 ± 4.1 years; 14.0 ± 0.2 years of education) participants were enrolled. All participants completed a 10‐min resting‐state functional magnetic resonance imaging scan to assess MTL dynamic network flexibility and two generalization tasks to assess cognition. Anthropometrics and aerobic fitness (via 6‐min walk test) were also determined. There was no difference in BMI (29.7 ± 6.4 vs. 31.9 ± 6.3 kg/m^2^, *p* = 0.083) or aerobic fitness (15.5 ± 2.6 vs. 15.1 ± 2.6 mL/kg/min; *p* = 0.445) between normotensive and hypertensive groups. However, normotensive participants had higher MTL dynamic network flexibility compared to hypertensive participants (0.42 ± 0.23 vs. 0.32 ± 0.25 mL, *p* = 0.040), and this was associated with higher mean arterial blood pressure (*r* = −0.21, *p* = 0.036). Therefore, hypertensive older African Americans demonstrated lower MTL dynamic network flexibility compared to their normotensive counterparts independent of BMI and aerobic fitness. Further studies are required to determine how blood pressure mediates AD risk in African Americans.

## 1 INTRODUCTION

The diagnoses of Alzheimer's disease and related dementia (ADRD) in the United States is nearly triple in African Americans compared with White Americans. The exact reason(s) is unclear, but this is likely due to in part higher rates of comorbidities including obesity and low levels of aerobic fitness (Koster et al., 2005; RTI International & Lines, 2014). Hypertension is also of particular interest since African Americans suffer from elevated blood pressure to greater extents than White Americans (Maraboto & Ferdinand, 2020; Musemwa & Gadegbeku, 2017), and this could contribute to increased pulsatile pressures to the brain that raise neuronal damage risk. Indeed, chronic high blood pressure (BP) has been noted to impair the structural and functional integrity of cerebral vasculature, thereby promoting microvascular rarefaction and dysfunction that leads to reductions in cerebral blood flow (Aronow, 2017; Canavan & O'Donnell, 2022; Ungvari et al., 2021). Furthermore, hypertension may disrupt the blood–brain barrier integrity, promote neuroinflammation, and contribute to amyloid deposition, which is a hallmark pathological marker of Alzheimer's disease (AD) (Canavan & O'Donnell, 2022; Ungvari et al., 2021). Together, these observations highlight understanding how hypertension impacts cerebrovascular disease is of clinical importance (Bezerra et al., 2012; Fanning et al., 2014; Knopman et al., 2010; Ungvari et al., 2021).

Recent brain imaging studies suggest that midlife hypertension is related to an increased cerebral amyloid burden as determined via positron emission tomography (Gottesman et al., 2017; Langbaum et al., 2012; Rodrigue et al., 2013). While promising evidence exists between midlife hypertension and ADRD, late‐life hypertension and heightened ADRD risk are inconclusive (Joas et al., 2012; Li et al., 2007; Skoog et al., 1996). One possibe theory for this discrepancy is that hemodynamic regulation may be altered by neurodegeneration years prior to disease onset (Lennon et al., 2019). To this end, most studies examining dementia risk use biomarkers (amyloid‐beta) and other diagnostic tools (composite cognitive measures), although it is not clear whether these outcomes are best for identification of AD/ADRD progression. Resting‐state functional magnetic resonance imaging (rs‐fMRI) in the medial temporal lobe (MTL) region in older adults has gained attention since it was validated for evaluating neural dysfunction in cognitively unimpaired at‐risk populations (Sinha et al., 2021). Moreover, MTL dynamic network flexibility may underlie the ability to apply previous learning to new situations and contexts, a hippocampal‐dependent cognitive function known as *generalization* (Myers et al., 2002, 2003, 2008; Sinha et al., 2020, 2021). Poorer generalization scores have also been linked to smaller MTL subregion volumes in cognitively unimpaired carriers of autosomal dominant AD genetic mutations (Petok et al., 2018). However, the role of hypertension in mediating alterations in MTL dynamic network flexibility in older African Americans is unknown. Therefore, we tested the hypothesis that hypertension would relate to lower MTL dynamic network flexibility as well as cognitive generalization (Rutgers Acquired Equivalence Task and Concurrent Discrimination and Transfer Task) independent of obesity and aerobic fitness.

## 2 MATERIALS AND METHODS

### 2.1 Participants

One hundred sixteen cognitively unimpaired older African Americans were recruited as part of the Rutgers University—Newark longitudinal *Pathways to Healthy Aging in African Americans* study (i.e., R01AG053961). Recruitment was from the greater Newark area and included flyers and word of mouth via senior centers, churches, medical centers, public and subsidized housing locations, and other community partners. Participants were included if they identified as Black or African American, were >60 years, and had a score a 24 or higher on the Mini‐Mental State Examination (MMSE), suggesting individuals had no cognitive impairments via an individual's orientation, registration, attention and calculation, recall, and language (Schultz‐Larsen et al., 2007). Participants were also required to pass the *Ishihara's Test for Color Deficiency* because the generalization tasks presented later in the session used colors as a visual cue. People were excluded if they had neurodegenerative diseases, learning disabilities, self‐reported abuse of alcohol and/or drugs, a medical procedure that required general anesthesia in the past 3 months, color blindness (the generalization tasks use colors as a visual cue), taking any medications typically prescribed for dementia, or contraindications (e.g., cardiac pacemaker, metallic stent, claustrophobia) to MRI. Subsequently, participants answered demographic questions and indicated their race/ethnicity, highest level of education, residency, and lifestyle behaviors including exercise and diet habits. Additionally, all female participants reported being post‐menopausal.

### 2.2 Hypertension assessment

Individuals were defined as hypertensive with systolic blood pressure (SBP) levels ≥130 mmHg (i.e., Stage 1) compared with those who were identified as normotensive <130 mmHg per the American Heart Association. Given SBP, compared diastolic blood pressure (DBP), has been linked to cognitive decline due to in part greater subcortical white matter lesion burden and genetic predisposition for ADRD by the *APOE ε4* allele (Daugherty, 2021), we defined hypertension herein via SBP. After 5‐min of quiet rest, blood pressure was assessed three times in a seated position using the automatic Microlife Blood Pressure Monitor (Microlife USA, Inc, Clearwater, FL) with proper cuff size. Data were averaged with 1‐min breaks in between. Participants were instructed not to cross legs and place feet on the floor. Pulse pressure (PP) was calculated as SBP–DBP. Mean arterial pressure (MAP) was also estimated as DBP × 1/3 (PP).

### 2.3 Anthropometrics

Baseline physical measurements included resting BP, height, and weight. Weight and height were assessed using the SECA 777 Digital Scale (SECA, Hamburg, Germany). Weight was measured to the nearest 0.1 kg. Height was measured to the nearest 0.1 cm. BMI was calculated as the participant's weight in kilograms divided by the square of height in meters.

### 2.4 Six‐min walking distance test (6MWDT)

The 6MWDT is a validated test of aerobic capacity that is used to reliably estimate maximal oxygen consumption (*V*O_2_max) (Rikli & Jones, 1998). From a standing start, participants were instructed to walk continuously back and forth between a 90‐foot course at the fastest pace they felt they could maintain throughout the duration of the examination in 6‐min. Participants were allowed to stop and stand or sit in a chair if needed and were instructed to walk again when they felt able. Distance walked was recorded at the end of the test to the nearest 0.1 m as the number of full and partial laps completed. This measure of aerobic capacity was then used to estimate the participant's *V*O_2_max using the following equation: *V*O_2_max = 4.948 + (0.023 × [total distance covered]) (Ross et al., 2010).

### 2.5 Rutgers generalization tasks

Individuals underwent two tests of cognitive function. First, participants underwent an *Acquired Equivalence Task* as previously described elsewhere (Myers et al., 2003). In brief, participants learned to form connections between faces and fish via trial‐and‐error learning. In each trial, participants saw a cartoon face and two fish with different colors and guessed which fish belongs to each face. During the training phase, participants learned to pair Face A with Fish 1 and Face B with Fish 1. In the process, participants should have learned that Face A and Face B are equivalent concerning the color of fish they are paired with. Next, participants learned to pair Face A with a new fish, Fish 2. During the final (generalization) phase, feedback was not given. Participants were tested on the transfer of their knowledge to novel pairings; if they previously learned that Face A and B were equivalent, then participants should have generalized that Face B is paired with Fish 2, even though they had not yet been presented with the pairing. Previous theoretical models demonstrated that acquired equivalence depends on MTL's entorhinal cortex (EC) and parahippocampal regions. Previous results suggest that participants with structural changes in the MTL associated with prodromal AD show normal learning but are selectively impaired in generalization. In contrast, abstraction is unimpaired in healthy aging (Myers et al., 2003, 2008).

Secondarily, people then underwent the *Concurrent Discrimination and Transfer Task* to assess ability to generalize past learning and apply these patterns to novel situations. Changes in the preclinical AD hippocampus and MTL circuits have revealed varying performance on this task (Myers et al., 2002, 2008) and this protocol has been described elsewhere (Myers et al., 2002, 2008; Sinha et al., 2020, 2021). Briefly, participants were presented with two phases to complete during the task: a training phase and a generalization phase. During the training phase, the participant was presented with a pair of objects on the screen. In the initial training phase trials, the pair of objects simply vary by shape, but remain the same color. Subsequently, participants learned to choose the appropriate objective pair. The variable of interest would thus be the number of errors committed during the transfer phase. Lower scores demonstrate better generalization performance.

### 2.6 MRI data acquisition

Neuroimaging scans were completed at the Rutgers University Newark Brain Imaging Center (RUBIC). Participants underwent MRI using a 3T Siemens Trio scanner equipped with a 32‐channel Multiband parallel encoding coil. They received a structural scan performed in the sagittal plane, which involved a high‐resolution 3D magnetization‐prepared rapid gradient echo (MP‐RAGE) sequence with the following scanning parameters: a repetition time (TR) of 1900 ms, an echo time (TE) of 2.52 ms, a 9‐degree flip angle, a voxel size of 1.0 × 1.0 × 1.2 mm, a field of view (FOV) of 270 × 254 × 212, 176 slices with no gap, and a total acquisition time of 9 min. High‐resolution multiband echo‐planar images were acquired with a FOV measuring 208 × 208 × 125. These images had a TE of 30 ms, an isotropic resolution of 1.8 mm, a TR of 664 ms, a flip angle of 30°, and a multiband acceleration factor of 5. A total of 45 axial slices were obtained, covering the entire brain. The utilization of the multiband parallel imaging allowed for the acquisition of high‐resolution functional images with rapid whole‐brain coverage. This approach facilitated the simultaneous acquisition of multiple slices, leading to a significant reduction in acquisition time and minimized distortion due to magnetic susceptibility. Also, this technique had improved temporal efficiency which provided greater statistical power (Feinberg et al., 2010).

### 2.7 rs‐fMRI: Preprocessing

The preprocessing and analysis of all neuroimaging data were conducted using Analysis of Functional NeuroImages (AFNI) software on Linux platforms. The standardized afni_proc.py pipeline was followed for the analyses. Data underwent several preprocessing steps, including slice timing correction (3dtshift), despiking (3dDespike), motion correction (3dvolreg), coregistration with T1‐weighted anatomical images (align_epi_anat.py), automated masking to exclude non‐brain voxels (3dautomask), and bandpass filtering within the 0.06–0.12 Hz frequency range. Trials with excessive motion (>0.3 mm) were excluded from the time series using a custom script. In accordance with the methods proposed (Power et al., 2012), the signal regression was performed on white matter and ventricular regions to account for scanner artifacts and motion‐related noise. This regression was carried out using ANATICOR (Jo et al., 2010), which estimated and applied ventricular signals and local white matter to nearby gray matter voxels. Functional scans were aligned with each subject's skull‐stripped MP‐RAGE (align_epi_anat.py). Voxel time courses were estimated through univariate regression (3dDeconvolve), incorporating nuisance variables for six motion parameters (pitch, roll, yaw, x, y, and z frame displacement) and linear scanner drift. To achieve subject‐specific alignment, Advanced Normalization Tools (ANTs) software was employed to warp participant structural scans into in‐house high‐resolution 0.65 mm isotronic templates using a diffeomorphic nonlinear registration algorithm (SyN) (Avants et al., 2011; Klein et al., 2009). The resulting transformation parameters were aligned with the custom template by being applied to the coplanar functional data obtained from the aforementioned regression.

### 2.8 rs*‐*fMRI: Dynamic network construction

**FIGURE 1:**
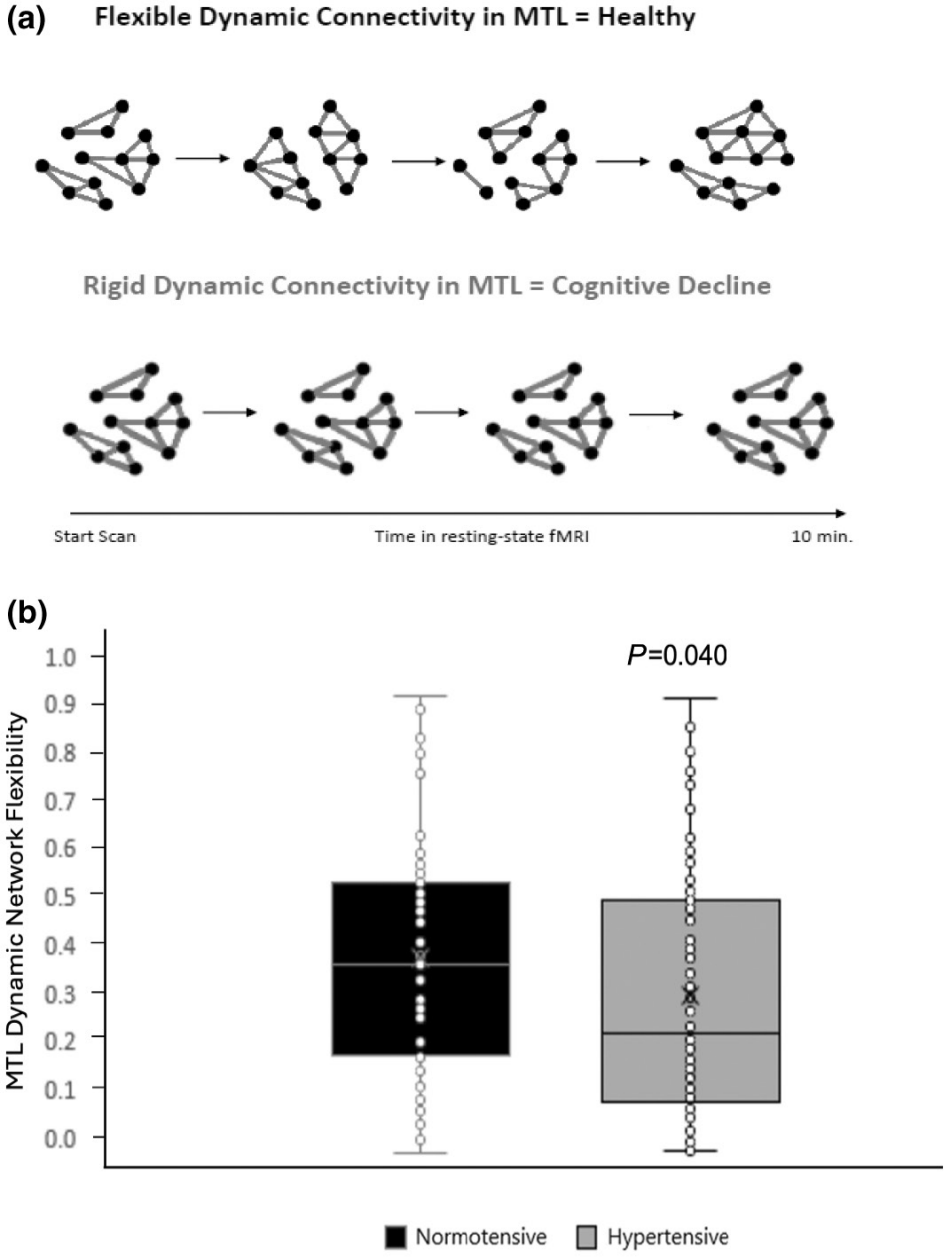
Resting‐state MTL dynamic network flexibility example (a) and comparison between normotensive and hypertensive participants (b). MTL, medial temporal lobe.

This protocol is discussed in detail elsewhere (Sinha et al., 2021). But, in brief, we investigated dynamic functional connectivity within the MTL, encompassing hippocampal subfields (subiculum, CA1, and DG/CA3), and cortical regions (perirhinal cortex, parahippocampal cortex, anterolateral EC, and posteromedial EC). Lateral and medial ECs provide input to the hippocampus, with the perirhinal and parahippocampal regions supplying input to the lateral and medial ECs, respectively. Functional distinctions between the lateral and medial EC have been demonstrated in studies in rodents and humans (Keene et al., 2016; Knierim et al., 2014; Reagh & Yassa, 2014), with recent evidence suggesting an anterolateral to posteromedial functional division in the human EC (Maass et al., 2015; Navarro Schröder et al., 2015). Accordingly, in our study, we treated the posteromedial and anterolateral ECs as separate regions of interest (ROIs) within the MTL network. Figure 1a depicts an example of rigid and dynamic connectivity within our network.

### 2.9 Statistics

SPSS (IBM, version 28.0) was used for statistical analysis. Data were assessed for normality using the Shapiro–Wilk test, and the Levene's test of homogeneity was used to test homogeneity of variance. Analysis of variance was used to analyze group differences, and age, sex, and education were included as co‐variates to isolate the effects of hypertension. Significance was accepted as *p* < 0.05. Cohen's *d* effect size was also calculated to estimate small (0.2), moderate (0.5), or large (0.8) physiologic relevance. Data are reported as mean, standard deviation, and percentages.

## 3 RESULTS

### 3.1 Demographics

Participants with hypertensive were slightly older than those with normotension (*p* < 0.006; Table 1). However, no significant differences between sex (% female), education, and MMSE scores were noted. There was also no significant difference in BMI or estimated *V*O_2_max (Table 1). As expected, only participants with hypertension reported taking BP medication (*n* = 35 of 79 or 44.3%).

**TABLE 1:** Participant characteristics.

	Normotensive	Hypertensive	*p‐*value
Demographics (*N*)	37	79	
Age (years)	64.4 ± 4.9	66.8 ± 4.1	0.006
Sex (female %)	82.5	79.5	0.921
Education (years)	14.3 ± 2.1	14.0 ± 0.2	0.521
MMSE	28.5 ± 1.5	28.1 ± 1.7	0.301
Anthropometrics and fitness			
BMI (kg/m^2^)	29.7 ± 6.4	31.9 ± 6.3	0.083
Mass (kg)	78.7 ± 15.9	86.6 ± 18.9	0.027
*V*O_2max_ (mL kg^−1^ min^−1^)	15.5 ± 2.6	15.1 ± 2.6	0.445
Blood pressure			
SBP (mmHg)	118.3 ± 10.8	151.5 ± 17.3	<0.001
DBP (mmHg)	73.8 ± 14.0	86.8 ± 10.4	<0.001
PP (mmHg)	44.5 ± 14.5	64.8 ± 15.1	<0.001
MAP (mmHg)	88.6 ± 11.1	108.3 ± 11.1	<0.001
Medication	0.0%	44.3%	

*Note*: Values are expressed as mean ± standard deviation. Hypertension is defined as ≥SBP 130 mmHg.

Abbreviations: BMI, body mass index; DBP, diastolic blood pressure; MAP, mean arterial pressure; MMSE, Mini‐Mental State Examination; PP, pulse pressure; SBP, systolic blood pressure; *V*O_2max_, maximal oxygen capacity.

### 3.2 Dynamic functional connectivity and cognition

Participants who were normotensive had significantly higher MTL dynamic network flexibility than those with hypertension (*p* = 0.040; *d* = 0.245; Figure 1b). Conversely, there were no significant differences between groups in the Rutgers Acquired Equivalence Task (*p* = 0.865; *d* = 0.252) or Concurrent Discrimination and Transfer Task (*p* = 0.396; *d* = 0.167).

### 3.3 Correlations

MAP was significantly associated with MTL dynamic network flexibility (*ρ* = −0.21 and 0.036; Table 2). No significant relationships were noted between SBP, DBP, PP, BMI, and *V*O_2_max with MTL dynamic network flexibility (*p* > 0.05). Additionally, no significant associations were identified between MAP, DBP, and PP and the Rutgers Acquired Equivalence Task or the Concurrent Discrimination and Transfer Task (*p* > 0.05).

**TABLE 2:** Correlations between blood pressure and cognitive assessments.

	MTL flex	Fish	Choose	SBP	DBP	MAP	PP	BMI	*V*O_2_max
MTL Flex	‐								
Fish	0.14	‐							
Choose	0.24**	0.15	‐						
SBP	−0.19	−0.01	−0.05	‐					
DBP	0.17	0.01	−0.04	0.64**	‐				
MAP	−0.21*	0.12	−0.05	0.88**	0.92**	‐			
PP	−0.04	0.03	−0.03	0.78**	0.09	0.43**	‐		
BMI	−0.01	−0.04	0.11	0.19*	−0.02	0.10	0.26**	‐	
*V*O_2_max	−0.04	0.09	0.01	−0.14	0.01	−0.08	−0.22*	−0.28	‐

* *p* < 0.050.

** *p* < 0.010.

Abbreviations: choose, concurrent discrimination and transfer task; DBP, diastolic blood pressure; Fish, acquired equivalence and generalization task; MAP, mean arterial pressure; MTL Flex, medial temporal network flexibility; PP, pulse pressure; SBP, systolic blood pressure.

## 4 DISCUSSION

The main finding was that participants who were normotensive displayed higher MTL dynamic network flexibility than individuals with hypertension. This was further corroborated by elevated MAP relating to low MTL dynamic network flexibility. Importantly, these results were independent of age, sex, education as well as BMI and aerobic capacity. Although no significant differences were observed between groups in either generalization tasks, this collective work suggests that hypertension may impact brain regions linked to AD/ADRD in older African American adults. Indeed, hypertension is considered a chief component in vascular aging and has been shown to be associated with neurodegenerative change before the onset of dementia (Nation et al., 2015). Our findings align with previous investigations examining reduced rs‐fMRI in hypertensive patients (Bu et al., 2018; Feng et al., 2020; Liao et al., 2023). In fact, one study identified reduced functional connectivity in the hippocampal regions of hypertensive participants who were comprised of mostly White individuals (Feng et al., 2020). However, they report that changes in functional connectivity mediated changes in lower prospective memory. Our current results did not demonstrate a reduction in generalization performance among older African Americans in the hypertensive BP ranges compared to non‐hypertensive counterparts. This may indicate our novel MTL dynamic network flexibility measure is sensitive to capturing early neural deficits in cognitively unimpaired hypertensive participants. Thus, these findings expand on current literature by characterizing the relations of hypertension in older African adults. Further, these findings may have clinical relevance at capturing early neural deficits in a preclinical population prior to decrements in cognition via generalization tasks.

There are a few possible reasons why hypertension was related to low MTL dynamic network flexibility. Vascular comorbidities such as obesity play a causal role in hypertension. In fact, 75% of hypertension cases can be attributed directly to obesity (Landsberg et al., 2013). In the current investigation, there were no significant differences in BMI between groups, which suggests excess adiposity is unlikely to explain the differences in dynamic network flexibility. It should be noted that approximately 84% of our sample was classified as overweight or obese. A consideration though is that this lack of relationship between obesity and network flexibility could relate to the fact that we did not measure body fat via DXA or air displacement. Further, we did not determine adipose‐derived inflammation, which has been linked to cognitive decline (Gómez‐Apo et al., 2021). It should be noted, however, that BMI is a valid population index of obesity and additional work is warranted. Another factor contributing to hypertension may be lower aerobic fitness levels. Aerobic fitness is mainly an indirect measure of cardiac output and arterial venous oxygen extraction. While oxygen extraction is often attributable to mitochondrial function, and mitochondrial dysfunction in the brain is tied to decline in cognitive function (Sharma et al., 2021; Skattebo et al., 2020), we did not observe a direct relationship between MTL dynamic network flexibility and aerobic fitness. While future studies using direct measures of aerobic fitness via indirect calorimetry are needed to confirm our results (Malin et al., 2022), it is worth acknowledging that blood pressure is reflective of cardiac output. Interestingly, we did detect a significant correlation between mean arterial blood pressure and MTL. This suggests factors related to mean arterial blood pressure could impact brain health.

Blood pressure is important for systemic and cerebral vascular blood flow (Son et al., 2015). Prior work reported that cerebral blood flow in the anterior cingulate cortex and the left posterior cingulate cortex was reduced in cognitively unimpaired hypertensive participants (Dai et al., 2008). Moreover, the SPRINT trial at baseline reported that hypertension reduced default mode network connectivity and higher white matter lesion volume both related to reduced cognitive function in cognitively unimpaired participants (Shah et al., 2021). Although we did not directly measure cerebral blood flow of vasculature structures in the brain, we report that higher MTL dynamic network connectivity was associated with lower MAP. In contrast, the lack of association between SBP and MTL dynamic network connectivity was somewhat surprising to observe in the present work. We do not have a readily available reason for this lack of association, but typically BP refers to pressure in the brachial artery. SBP specifically is linked to pressure exerted by the heart to overcome resistance in the periphery. MAP, however, is the average BP that promotes blood flow. Taken together, our work suggests that the higher MAP may be required to promote blood flow in individuals with hypertension, thereby raising the risk of burden on end‐organ microvasculature structures.

It is important to note that we did not detect differences in cognitive tasks between hypertensive and normotensive older African Americans. This observation is not necessarily surprising given we enrolled participants who were cognitively normal via the MMSE. Declines in neurologic function have been suggested to occur up to approximately 15–20 years before any noticeable cognitive impairments (Kremen et al., 2014). This in part helps explain why midlife hypertension elevates risk for cognitive decline. However, while our adults studied were older, it is possible that the present study findings suggest neural changes could precede cognitive abilities. Thus, the findings could have physiologic relevance as they illuminate how hypertension could impact brain regions that are associated with cognitive/memory decline as seen in those with AD/ADRD (Alzheimer's Association, 2019; Gottesman et al., 2017; Lennon et al., 2019). Furthermore, the literature is mixed on the exact role hypertension plays on cognition as some show relations (Gottesman et al., 2017; Langbaum et al., 2012; Lennon et al., 2019; Rodrigue et al., 2013; Shah et al., 2012), whereas others do not (Joas et al., 2012; Li et al., 2007; Skoog et al., 1996). In fact, some have even reported that lower global cognitive outcomes were paralleled with lower DBP, but higher SBP and PP (Levine et al., 2019). Taken together, these findings collectively demonstrate that future investigations should consider examining regional blood flow and white matter lesion volume in ROIs in parallel with MTL dynamic network flexibility measures to enhance understandings of neural decline in older African American adults.

This study is not without limitations. Associations do not equal causation and additional mechanistic studies are needed to understand how hypertension relates to altered neural flexibility. In fact, we acknowledge that it is possible that changes in brain function of the hippocampus and other regions (e.g., insular cortex, pons, and medulla) could impact blood pressure regulation (Burke et al., 1994). Further, our participant sample was recruited from the same metropolitan region and was mostly female. We also did not collect detailed information as to dose, frequency or medication type to delineate the impact of anti‐hypertensive treatment on respective outcomes. As a result, we cannot generalize these findings across geographic regions whereby social determinants may influence physiologic underpinnings of cognition and neural adaptations for brain health, nor can we say definitively whether these results are comparable in other races/ethnicities. In addition, we recognize that some outcomes had small effect sizes between hypertensive and normotensive groups. This might reflect that underlying issues such as arterial stiffness and/or endothelial function are more intimately linked to changes in cerebral vascular function and neural networks than blood pressure per se. Thus, future studies should consider such vascular assessments in the periphery to discern influence on brain function. In this cohort, we were also unable to assess genetic risk for neural alterations, and additional investigations should consider genetic interactions that may amplify the risk for cognitive decline in African Americans such as APOL1, APOE‐*ε4*, and ABCA7. Nevertheless, since only 5–10% of ADRD and cognitive‐based research is in African Americans (Alzheimer's Association, 2019), these work add to the existing literature.

In conclusion, the findings of our current investigation suggest reduced MTL dynamic network flexibility in older hypertensive African Americans independent of medication, obesity, or aerobic fitness level. Although there was no difference in generalization tasks, these findings suggest that older African Americans with hypertension could be at risk for MTL‐based cognitive decline. Future work ought to consider assessments of cerebral blood flow in the MTL to understand the mechanistic action of our current findings, as well as assess social determinants of health to provide insight on personalized interventions that reduce hypertension‐mediated cognitive decline risk.

## FUNDING INFORMATION

This work was supported by the National Institutes of Health‐National Institute on Aging (Grant Numbers R01AG053961 and R01AG078211), Office of Minority Health at U.S. Department of Health and Human Services (Grant Number MH‐STT‐15‐001), the NJ Department of Health (Grant Number OMMH21HDP002), and by the Chancellor's and Provost offices at Rutgers University‐Newark.

## CONFLICT OF INTEREST STATEMENT

Authors decline no conflict of interest.

## ETHICS STATEMENT

Participants provided written informed consent, as ethics approval was obtained from the Rutgers University Institutional Review Board. All methods were performed in accordance with guidelines and regulations of the Declaration of Helsinki.

## Data Availability

Data will be made available upon reasonable request to the corresponding author.
